# Multivariable mortality risk prediction using machine learning for COVID-19 patients at admission (AICOVID)

**DOI:** 10.1038/s41598-021-92146-7

**Published:** 2021-06-17

**Authors:** Sujoy Kar, Rajesh Chawla, Sai Praveen Haranath, Suresh Ramasubban, Nagarajan Ramakrishnan, Raju Vaishya, Anupam Sibal, Sangita Reddy

**Affiliations:** 1 Apollo Hospitals, Jubilee Hills, Hyderabad, 500033 India; 2 Indraprastha Apollo Hospitals, Sarita Vihar, New Delhi, India; 3 Apollo Multispecialty Hospitals, Kolkata, 700054 India; 4 Apollo Hospitals, Greams Road, Chennai, India; 5 Indraprastha Apollo Hospitals, Sarita Vihar, New Delhi, India

**Keywords:** Machine learning, Risk factors, Viral infection

## Abstract

In Coronavirus disease 2019 (COVID-19), early identification of patients with a high risk of mortality can significantly improve triage, bed allocation, timely management, and possibly, outcome. The study objective is to develop and validate individualized mortality risk scores based on the anonymized clinical and laboratory data at admission and determine the probability of Deaths at 7 and 28 days. Data of 1393 admitted patients (Expired—8.54%) was collected from six Apollo Hospital centers (from April to July 2020) using a standardized template and electronic medical records. 63 Clinical and Laboratory parameters were studied based on the patient’s initial clinical state at admission and laboratory parameters within the first 24 h. The Machine Learning (ML) modelling was performed using eXtreme Gradient Boosting (XGB) Algorithm. ‘Time to event’ using Cox Proportional Hazard Model was used and combined with XGB Algorithm. The prospective validation cohort was selected of 977 patients (Expired—8.3%) from six centers from July to October 2020. The Clinical API for the Algorithm is http://20.44.39.47/covid19v2/page1.php being used prospectively. Out of the 63 clinical and laboratory parameters, Age [adjusted hazard ratio (HR) 2.31; 95% CI 1.52–3.53], Male Gender (HR 1.72, 95% CI 1.06–2.85), Respiratory Distress (HR 1.79, 95% CI 1.32–2.53), Diabetes Mellitus (HR 1.21, 95% CI 0.83–1.77), Chronic Kidney Disease (HR 3.04, 95% CI 1.72–5.38), Coronary Artery Disease (HR 1.56, 95% CI − 0.91 to 2.69), respiratory rate > 24/min (HR 1.54, 95% CI 1.03–2.3), oxygen saturation below 90% (HR 2.84, 95% CI 1.87–4.3), Lymphocyte% in DLC (HR 1.99, 95% CI 1.23–2.32), INR (HR 1.71, 95% CI 1.31–2.13), LDH (HR 4.02, 95% CI 2.66–6.07) and Ferritin (HR 2.48, 95% CI 1.32–4.74) were found to be significant. The performance parameters of the current model is at AUC ROC Score of 0.8685 and Accuracy Score of 96.89. The validation cohort had the AUC of 0.782 and Accuracy of 0.93. The model for Mortality Risk Prediction provides insight into the COVID Clinical and Laboratory Parameters at admission. It is one of the early studies, reflecting on ‘time to event’ at the admission, accurately predicting patient outcomes.

## Introduction

The current COVID-19 pandemic caused by SARS-CoV-2 is associated with high mortality and morbidity^1^. In India, over 10 million individuals have been affected by the virus (till mid January), with over 150 thousand people losing their lives, at a mortality rate of 1.44%^2^. However, the 30 days mortality rates at tertiary care hospitals in the US are far higher at 9.06% to 15.65%^3^. Various studies have been conducted to determine the mortality risk factors in COVID 19^4–6^. Understanding the clinical and laboratory predictors at admission can lead to appropriate determinants of mortality and improve triaging, bed and resource allocation, and improved patient management throughout health systems.

The datasets of COVID-19 patients can be integrated and analysed by Machine Learning (ML) algorithms to improve diagnostic speed and accuracy better and potentially identify the most susceptible people based on personalized clinical and laboratory characteristics^7^. These methods activate early insights of patient’s outcome with the predictors at the time of admission. Existing studies have used Machine Learning Algorithms (MLA) to determine COVID-19 mortality^8–10^.

**Figure 1:**
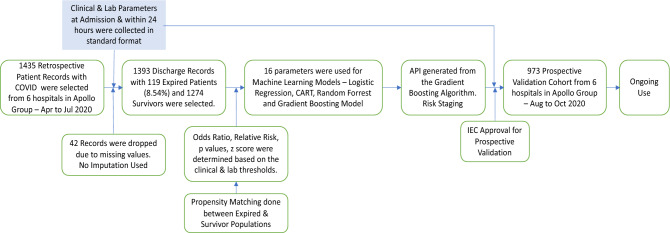
Schematic flow of the development and validation of the AICOVID algorithm to predict the risk of mortality at admission.

Due to the absence of similar studies in the Indian population, this research work was undertaken to develop and validate MLA based on the anonymized clinical and laboratory data to predict the outcome (Expired or Recovered) from retrospective evaluation of patients admitted with COVID (Fig. 1). Additionally, the algorithm determines the probability (risk) of Events (defined as Death or Expiry of Subjects), predicting mortality at 7 and 28 days. Secondarily this would provide clinical insights on various clinical and laboratory parameters. These are clinically and statistically relevant and help develop a Clinical API (application and programming interface) tool used by clinicians taking care of admitted patients even in low-cost settings.

The main contribution of the paper is to identify the risk factors for mortality at admission of COVID-19 patients and to build a multivariable Machine Learning model which would help in triaging, allocating resources, putting patients into an appropriate clinical protocol, and patient and family education at admission.

The remainder of this paper includes Methodology, Study design and modelling in “Methodology” section; “Results” in “Results” section, Interpretation and Limitations in “Limitation” section, followed by Conclusion in “Conclusion” section.

## Methodology

This study is designed as a multicenter, retrospective—prospective, observational, non-interventional study.

### Source of data

The retrospective data (for the development cohort) is collected from the anonymized clinical and laboratory records at admission from the discharge summaries of the patients and a standardized template (Annexure 1) from six Apollo Hospitals in India for the period of April to June 2020. These Hospitals are from Bangalore Chennai, Delhi, Hyderabad, Kolkata, and Navi Mumbai. On the prospective validation arm, the data elements at admission were provided by the site investigator in the electronic template (API—Fig. 4). Additional data were collected from discharge summaries, and the standardized template described. For discharged summaries, we used appropriately coded ICD-10 diagnosis data. The validation cohort was collected from the same six hospitals in the period between August to October 2020.

### Participants

The participants were admitted to the hospital with symptoms and history suggestive^11,12^ of Coronavirus Disease (COVID) with subsequent laboratory confirmation through Reverse Transcription Polymerase Chain Reaction (RT-PCR tests). The data were collected during admission at Emergency Room and following admission (within 24 h). The eligibility criteria for inclusion of the patients included—(a) patients presenting with COVID-19 related symptoms (with or without a history of contact/travel to geographical hot spots in the community), (b) subjects who comply with category B2/C in the Apollo Hospitals COVID protocol (See Annexure 2). The exclusion criteria were (a) patients admitted for other disease conditions and were subsequently found to have COVID during the hospital stay. The study did not include any specific intervention or treatment provided to the patient during admission or within its 24 h. The Institutional Ethics Committees of the six Apollo Hospitals (specified above) have approved the research and confirmed that all research was performed in accordance with relevant guidelines/regulations, and that informed consent was obtained from all participants and/or their legal guardians. Research involving human research participants has been performed in accordance with the Declaration of Helsinki.

### Outcome

The study's primary outcome was to develop comparable models with improved accuracy parameters, which would yield a risk predictor for mortality (in the next 7 and 28 days) at admission. Each predictor (clinical and laboratory variables) are studied for their odds and hazard ratios.

### Predictors

The Clinical Variables included patient’s basic information, including Age and Gender, Exposure and Travel history, and the number of days of symptoms before admission. It also included different symptoms like fever, cough, respiratory distress (shortness of breath), etc. The history of associated chronic diseases like diabetes, hypertension, heart and kidney disease, cancer, etc., was captured. Vitals at admissions like temperature, oxygen saturation, rate of respiration (ROR), and pulse rate were noted. Laboratory parameters were categorized into (a) Complete Hemogram including Red Cell Distribution Width, (b) Basic biochemistry including Kidney and Liver Profile Tests, (c) Coagulation Profile including Prothrombin Time, International Randomized Ratio (INR), Activated Prothomboplastin Time (APTT) and D-dimer, (d) Inflammatory markers like C-Reactive Protein (CRP), Lactic Acid Dehydrogenase (LDH), Ferritin, Interleukin-6 and Procalcitonin, were evaluated. Out of more than 65 predictors, we selected only 23 predictors based on their odds ratio, *p* value (Table 1), and clinical relevance. D-dimer and Interleukins were not selected in the study due to the smaller sample size (< 30%). For Validation data, there were no differences from the development data in setting, eligibility criteria, outcome and predictors.

**Table 1:** 23 key clinical and lab variables for the prediction of mortality at admission with COVID-19.

Development								Validation					
Clinical and lab parameters	Threshold	Recovered		Expired		Odds ratio		Recovered		Expired		Odds ratio	
		Mean	CI	Mean	CI	Odds ratio	*p* value	Mean	CI	Mean	CI	Odds ratio	*p* value
Age	60	47.19	46.28–48.09	61.13	57.98–64.28	4.59	< 0.0001	53.4	52.39–54.4	67.41	64.52–70.3	5.12	< 0.0001
Gender (male)	N/A	0.68	0.65–0.7	0.78	0.7–0.85	1.67	0.0328	0.72	0.69–0.75	0.85	0.77–0.93	1.19	0.2937
Fever	N/A	0.62	0.59–0.65	0.76	0.68–0.84	1.94	0.0043	0.58	0.55–0.62	0.02	0.01–0.06	0.17	< 0.0001
Cough	N/A	0.37	0.34–0.39	0.43	0.34–0.52	1.33	0.1641	0.5	0.47–0.53	0.47	0.36–0.58	0.74	0.0389
Respiratory distress	N/A	0.21	0.18–0.23	0.57	0.48–0.66	5.01	< 0.0001	0.13	0.1–0.15	0.51	0.4–0.62	3.08	< 0.0001
Weakness	N/A	0.09	0.08–0.11	0.17	0.1–0.24	1.96	0.0148	0.15	0.12–0.17	0.21	0.12–0.3	0.65	0.038
Diabetes	N/A	0.29	0.26–0.31	0.57	0.48–0.66	3.33	< 0.0001	0.39	0.36–0.42	0.52	0.41–0.63	2.15	< 0.0001
Hypertension	N/A	0.3	0.27–0.32	0.5	0.4–0.59	2.37	< 0.0001	0.42	0.39–0.45	0.58	0.47–0.69	2.09	< 0.0001
Chronic kidney disease	N/A	0.05	0.04–0.07	0.16	0.09–0.23	3.24	< 0.0001	0.05	0.04–0.06	0.19	0.1–0.27	4.1	< 0.0001
Chronic liver disease	N/A	0.01	0–0.01	0.02	0.01–0.04	3.44	0.1261	0.01	0–0.01	0.01	0.01–0.04	3.85	0.0378
Heart disease	N/A	0.06	0.05–0.07	0.24	0.16–0.32	5.04	< 0.0001	0.09	0.07–0.11	0.02	0.01–0.06	2.11	0.0017
Respiration rate	24	21.63	21.44–21.83	27.33	26.12–28.55	6.77	< 0.0001	21.56	21.29–21.82	28.49	26.74–30.25	4.56	< 0.0001
Oxygen saturation	0.88	0.97	0.96–0.98	0.88	0.86–0.9	22.32	< 0.0001	0.95	0.95–0.96	0.85	0.82–0.88	7.58	< 0.0001
WBC count	8000	6684	6510–6858	9907	8039–11,775	3.64	< 0.0001	7076	6596–7556	12,919	10,747–15,091	3.29	< 0.0001
Lymphocyte%	10	25.37	24.74–26	10.06	8.48–11.64	11.93	< 0.0001	30.63	29.86–31.4	19.64	17.31–21.98	3.92	< 0.0001
INR (PT)	1.3	1.13	1.12–1.15	1.32	1.24–1.39	4.58	< 0.0001	1.41	1.36–1.45	1.98	1.71–2.25	3.49	< 0.0001
Creatinine	1.3	1.19	1.11–1.28	1.79	1.43–2.15	3.26	< 0.0001	9.69	9.08–10.3	3.28	2.77–3.8	14.08	< 0.0001
Albumin	3.5	4.05	4.02–4.08	3.61	3.48–3.74	3.95	< 0.0001	4.08	4.05–4.11	3.62	3.43–3.81	3.1	< 0.0001
AST (aspartate aminotransferase)	60	47.66	45.54–49.79	64.54	48.34–80.74	1.58	0.0452	50.18	46.17–54.18	167.16	113.39–220.93	3.49	< 0.0001
Lactate dehydrogenase	450	315.07	307–322	565.79	509.5–622.0	8.6	< 0.0001	334.61	324.21–345	602.49	545.26–659.73	8.46	< 0.0001
Ferritin	800	518.98	446–591	1566.7	942.9–2190.4	3.76	< 0.0002	536.44	489–583	1772.16	1397.–2147	4.15	< 0.0001
C-reactive protein	48	35.72	28.9–42.54	145.92	87.5–204.34	5.35	< 0.0001	36.94	33.55–40.33	135.9	110.33–161.48	3.44	< 0.0001
Red cell distribution width	14.5	14.27	14.18–14.37	14.55	14.22–14.87	1.21	0.368	14.5	14.37–14.63	16.44	15.84–17.05	3.06	< 0.0001

### Sample size

The study included a total population of 2370 patients, including 1393 in the Development Cohort and 977 in the Validation cohort. The sample size was determined using an estimated 10 million COVID Cases in India (October 2020) at 95% confidence level and confidence interval 2.

### Missing data

The initial development cohort was 1435. Forty-two patient's data were dropped owing to missing fields. No imputations were used in the development or validation cohort. As described earlier, certain laboratory predictors were not selected due to the smaller sample size and missing values.

### Statistical analysis and modelling approach

The clinical and laboratory parameters were selected based on the odds ratios of the initial cohort. The parameters were subsequently run through the Propensity Matching for the binary classification of Event (Death—1) and Non-Events (Survival—0). The population is randomly divided into training (70%) and test (30%) in the development model. The 23 parameters were then put through the three models for maximizing the K-fold cross-validation AUC (Area Under Curve) using Python (3.7) to determine the performance of logistic regression, random forest models, and eXtreme gradient boosting (XGB) algorithm.

However, we considered the XGB (eXtreme Gradient Boosting) model, as the function of this model is an approximation of the data distribution considering the errors:

$${\mathrm{y}}_{\mathrm{i}}={\mathrm{F}}_{1}\left({\mathrm{x}}_{\mathrm{i}}\right)+{\mathrm{error}}_{1\mathrm{i}}$$

where ${\mathrm{y}}_{\mathrm{i}}$ is the predicted value and ${\mathrm{x}}_{\mathrm{i}}$ are the input values. ${\mathrm{F}}_{1}({\mathrm{x}}_{\mathrm{i}})$ is a function, and the relationship between $\mathrm{x}$ and $\mathrm{y}$ is not fully described.

1. We initialize the model by solving the following equation for the 23 input parameters:
  ${\mathrm{F}}_{0}(\mathrm{x})=\mathrm{argmin}\,{\sum }_{\mathrm{i}=1}^{\mathrm{n}}\mathrm{L}({\mathrm{y}}_{\mathrm{i}},\mathrm{\gamma }$); then we get
  $${\mathrm{F}}_{0}\left(\mathrm{x}\right)=\frac{{\sum }_{\mathrm{i}=1}^{\mathrm{n}}{\mathrm{y}}_{\mathrm{i}}}{\mathrm{n}}$$
  where $\mathrm{n}$ is the total number of observation, i.e., 1393. ${\mathrm{F}}_{1}({\mathrm{x}}_{\mathrm{i}})$ function is a weak learner, and the relationship between X and y is not fully described.
2. For no of iterations—m = 1 to M
  Gradient with respect to predicted value,
  $${\mathrm{r}}_{\mathrm{im}}=\,-\left\{\,\frac{\partial \mathrm{L}\left[{\mathrm{y}}_{\mathrm{i}},{\mathrm{F}}_{\mathrm{m}-1}\left({\mathrm{x}}_{\mathrm{i}}\right)\right]}{\partial {\mathrm{F}}_{\mathrm{m}-1}\left({\mathrm{x}}_{\mathrm{i}}\right)}\right\},$$
  where $\mathrm{i}$ is the index for observations, $\mathrm{m}$ represents the number of iterations with m ∈ [1, M]. M is the maximum number of iterations. L stands for loss functions.
3. Fit the weak learner ${\mathrm{h}}_{\mathrm{m}}(\mathrm{x})$ to the residuals by:
  Computing the ${\mathrm{\gamma }}_{\mathrm{m}}$ to solve the optimization problem:
  $${\mathrm{\gamma }}_{\mathrm{m}}=\mathrm{arg}\,\mathrm{min}\sum _{\mathrm{i}=1}^{\mathrm{n}}\mathrm{L}[{\mathrm{y}}_{\mathrm{i}},{\mathrm{F}}_{\mathrm{m}-1}\left({\mathrm{x}}_{\mathrm{i}}\right)+.{\mathrm{F}}_{\mathrm{m}}({\mathrm{x}}_{\mathrm{i}})]$$
  By solving this equation we can get:
  $${\mathrm{\gamma }}_{\mathrm{m}}=\,\frac{{\sum }_{\mathrm{i}=1}^{\mathrm{n}}{\mathrm{h}}_{\mathrm{m}}({\mathrm{x}}_{\mathrm{i}})\cdot [{\mathrm{y}}_{\mathrm{i}}-{\mathrm{F}}_{\mathrm{m}-1}\left({\mathrm{x}}_{\mathrm{i}}\right)]}{{{\sum }_{\mathrm{i}=1}^{\mathrm{n}}{\mathrm{h}}_{\mathrm{m}}({\mathrm{x}}_{\mathrm{i}})}^{2}}$$
4. Update the $${\mathrm{F}}_{\mathrm{m}}\left(\mathrm{x}\right)=\,{\mathrm{F}}_{\mathrm{m}-1}\left(\mathrm{x}\right)+\,{\mathrm{\gamma }}_{\mathrm{m}}\cdot {\mathrm{h}}_{\mathrm{m}}(\mathrm{x})$$
  squared error is used as the loss function, and the gradient of the loss function can be calculated as follows:
  $$\begin{aligned}{\mathrm{r}}_{\mathrm{im}}&=\,-\left\{\,\frac{\partial \mathrm{L}\left[{\mathrm{y}}_{\mathrm{i}},{\mathrm{F}}_{\mathrm{m}-1}\left({\mathrm{x}}_{\mathrm{i}}\right)\right]}{\partial {\mathrm{F}}_{\mathrm{m}-1}\left({\mathrm{x}}_{\mathrm{i}}\right)}\right\},\\ &=\,-\,\frac{\partial \{\frac{1}{2}\times [{\mathrm{F}}_{\mathrm{m}-1}\left({\mathrm{x}}_{\mathrm{i}}\right)-{\mathrm{y}}_{\mathrm{i}}{]}^{2}\}}{\partial {\mathrm{F}}_{\mathrm{m}-1}({\mathrm{x}}_{\mathrm{i})}}\\ &=-\frac{\partial \left\{\frac{1}{2}\times \left[{\mathrm{F}}_{\mathrm{m}-1}{\left({\mathrm{x}}_{\mathrm{i}}\right)}^{2}+{\mathrm{y}}_{\mathrm{i}}^{2}-2\times {\mathrm{F}}_{\mathrm{m}-1}\left({\mathrm{x}}_{\mathrm{i}}\right)\cdot {\mathrm{y}}_{\mathrm{i}}\right]\right\}}{\partial {\mathrm{F}}_{\mathrm{M}-1}\left({\mathrm{x}}_{\mathrm{i}}\right)}\\ &={\mathrm{y}}_{\mathrm{i}}-{\mathrm{F}}_{\mathrm{m}-1}\left({\mathrm{x}}_{\mathrm{i}}\right)\,\mathrm{for}\,\mathrm{i}=\mathrm{1,2},\dots \mathrm{n}.\end{aligned}$$

Python language is used to code the program. Python ML packages were used, namely Sklearn, numpy and pandas library is used for this work. The code snippet for XGB model = XGB Classifier (n estimators = 1997, learning rate = 0.2, max depth = 5, random state = 42). These details are obtained through multiple trial and error methods with many hyperparameters derived from the variables. Similarly for Random Forest Model n_estimators were selected as 100, with max depth at 5.

After training model and successful testing, the model is pickled and saved. This pickled model is hosted and served as the back end for requests from the front end user. The user's values will act as input for the model, and the predicted response would be output.

### Risk stratification

On the input of the individual data to the algorithm, the machine returns the value in percentage of the risk of mortality in the next 7 and 28 days. The risk thresholds between 0 and 15% are associated with low mortality rates (< 1%), while the moderate risk category 15–30% had 1–5% Mortality and high-risk category (> 30%) at > 5% Mortality. Further, the Cox Proportional Hazard model's addition returned the probability of mortality in 7 and 28 days and has been used to display in the output.

### Predictors analysis

Hazard Ratios are calculated for each predictor, based on their accepted clinical and laboratory thresholds, as applicable. (see Table 1 for Thresholds) Kaplan Meir curves and Violin plots were used to analyze the effect of individual variables on overall mortality.

### Performance evaluation

All models are evaluated based on their ability to discriminate between outcomes for the development cohort and the XGB Model for the validation cohort with the corresponding confidence intervals (CI). The AUC, accuracy, sensitivity—specificity, precision, predictive value, and likelihood ratios are computed for validation cohort with a standard threshold. Thresholds are derived through the mean cut off values of different parameters including their clinical and laboratory ranges.

### Patient and public involvement

The study has been conducted with retrospective data from April to July 2020, and hence development of the research question and outcome measures were not directly communicated to the patients in the development cohort. However, in the validation cohort, they were informed about the study design and outcome.

## Results

### Clinical and laboratory variables at admission (participants)

**Figure 2:**
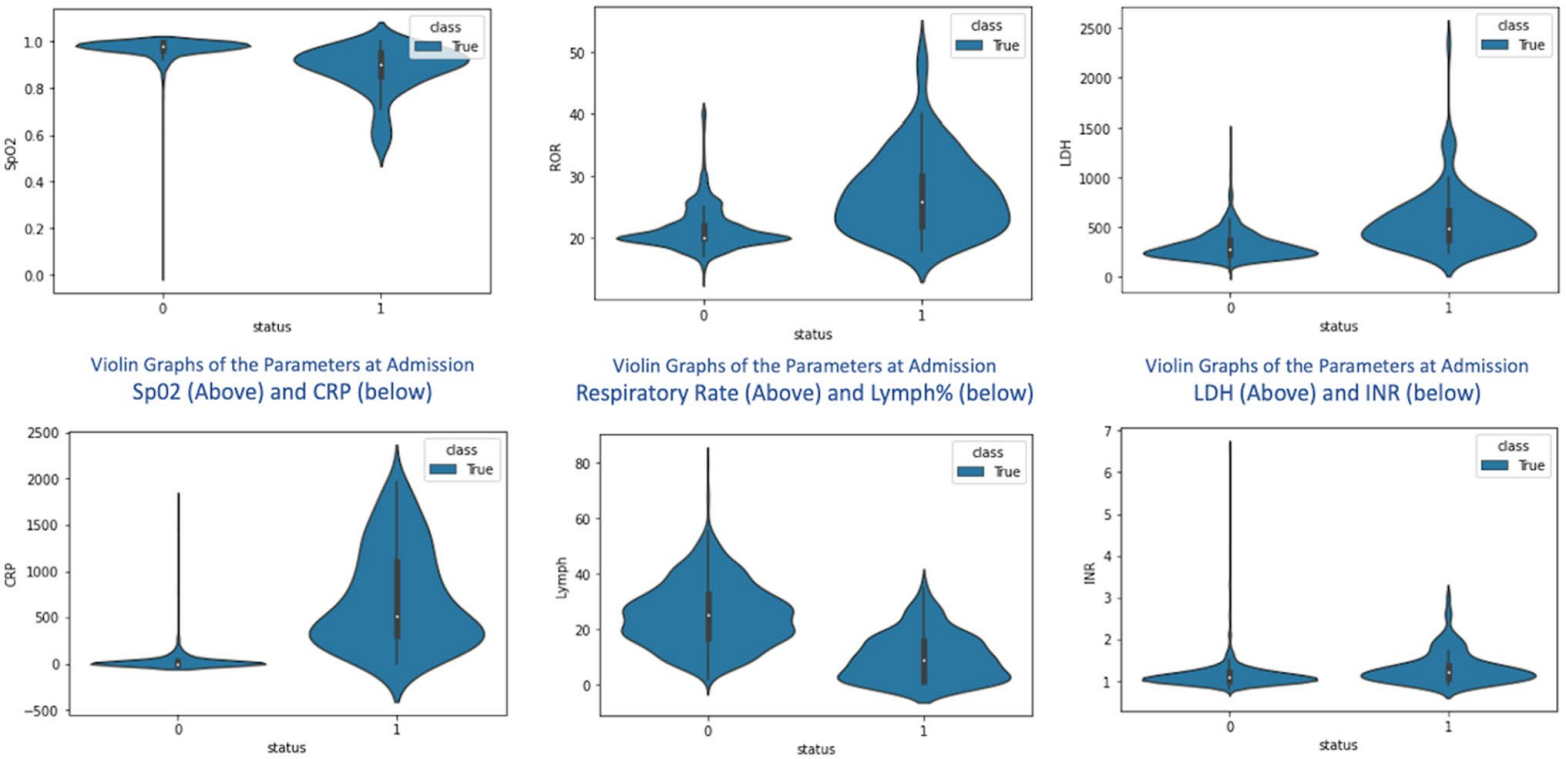
Combination of Box Plots and Kernel Density Plots reflect on different clinical and lab variables at admission and their representation for Survival (0) vs. Expired Patients (1). The white dot represents the median, the thick grey bar in the centre represents the interquartile range, the thin grey line represents the rest of the distribution, except for points that are outliers. [Development Cohort].

The average observed mortality rates are 8.54% (N = 1393) in the development cohort and 8.3% in the validation cohort (N = 977) for six different hospitals in April to July 2020 and August to October 2020, respectively. The average Length of Stay in the study population is 10.04 (9.75–10.3) days for survived patients and 12.32 (11.2–13.5) days for expired patients. In Age comparison, expired patients were older (mean age 61.1 vs. 47.2 in the development cohort and 67.4 vs. 53.4 in the validation cohort). The gender of expired patients was 78% & 85% male in the development and validation cohort. Further details of the symptoms, comorbidities, vitals at admission, and laboratory parameters are provided in Table 1. Significantly, the odds ratios for Respiratory Distress [5.01], Diabetes [3.33], Heart Disease [5.04] are high in the development cohort as symptoms and comorbidities. Odds ROR > 24/min, Oxygen saturation < 90% are at 6.77 and 22.32 respectively. In lab parameters, Lymphocytes below 10% [11.93], Lactate Dehydrogenase > 250 units [8.6] and C-reactive protein > 48 units [5.35] show high significance. Figure 2—shows the different parameters with violin graphs.

### Performance indicators (model development and validation)

**Figure 3:**
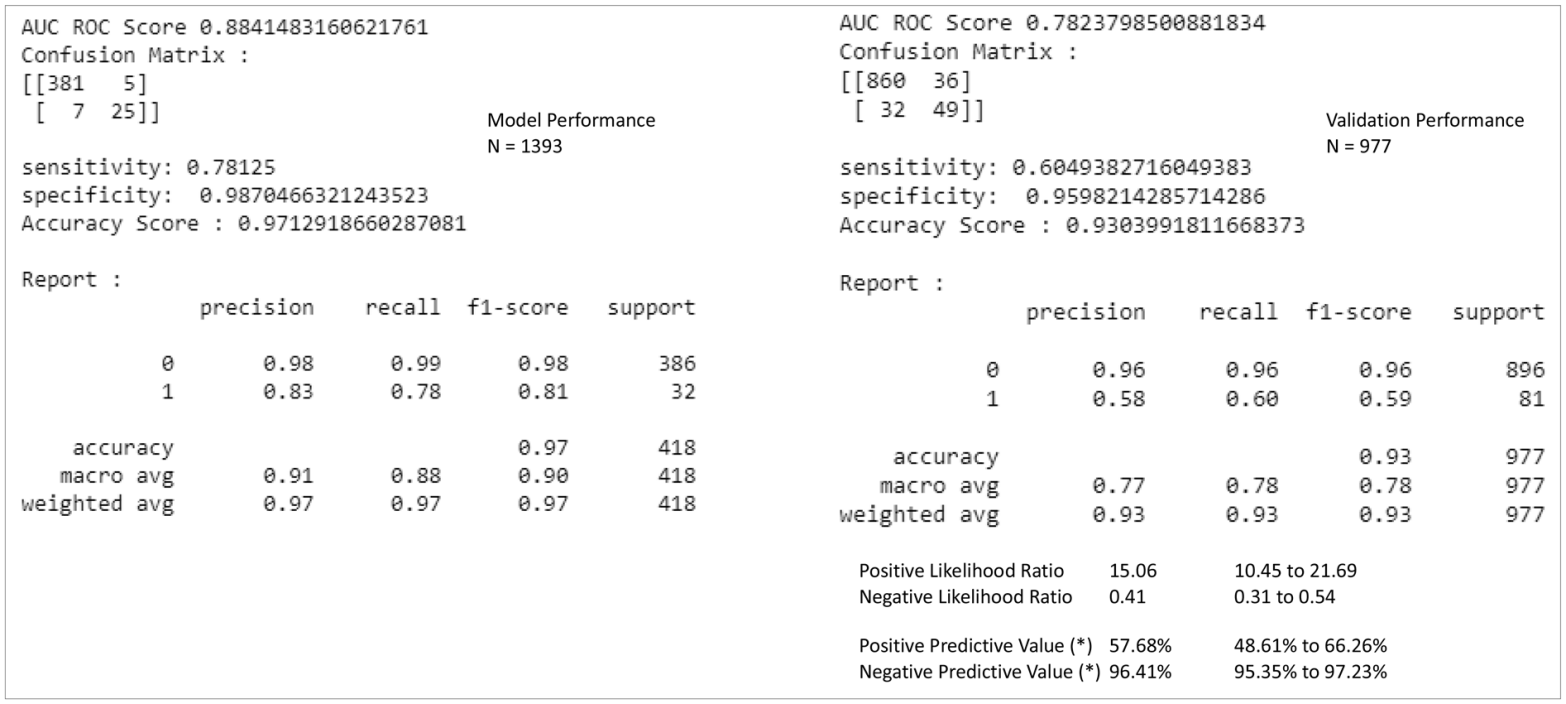
Performance of eXtreme Gradient Boosting (XGB) Algorithm for the Development and Validation Cohort.

The performance of the XGB model is summarized in Fig. 3 below. The AUC for the developed algorithm is 0.88, Accuracy score at 0.97, and precision at 0.91. The validation cohort's performance is AUC at 0.78, Accuracy score at 0.93, and precision at 0.77. The positive likelihood ratio is at 15.06, and the negative predictive value is 96.41%. The critical clinical and laboratory variables in the ML model depict the important predictors of the overall outcome and interplay between each of them.

### Propensity matching scores

The results of the propensity matching scores for the Survivor and Mortality Groups in the development dataset using Hosmer and Lemeshow Goodness of Fit (GOF) test are—X-squared (1.2341), *df* (8), *p* value (0.9963), AIC value (241.148), Log-likelihood (− 94.574), adjusted R square (0.7509801), Pseudo-R-squared values at—McFadden (0.750980), Nagelkerke (Cragg and Uhler) (0.799456). We achieved following values for the validation set—X-squared (3.2107), *df* (8), *p* value (0.9204), AIC value (288.9656), Log-likelihood (− 118.4828), adjusted R square (0.6598251), Pseudo-*R* squared values at McFadden (0.659825), Nagelkerke (Cragg and Uhler) (0.740693).

### Comparison

**Figure 4:**
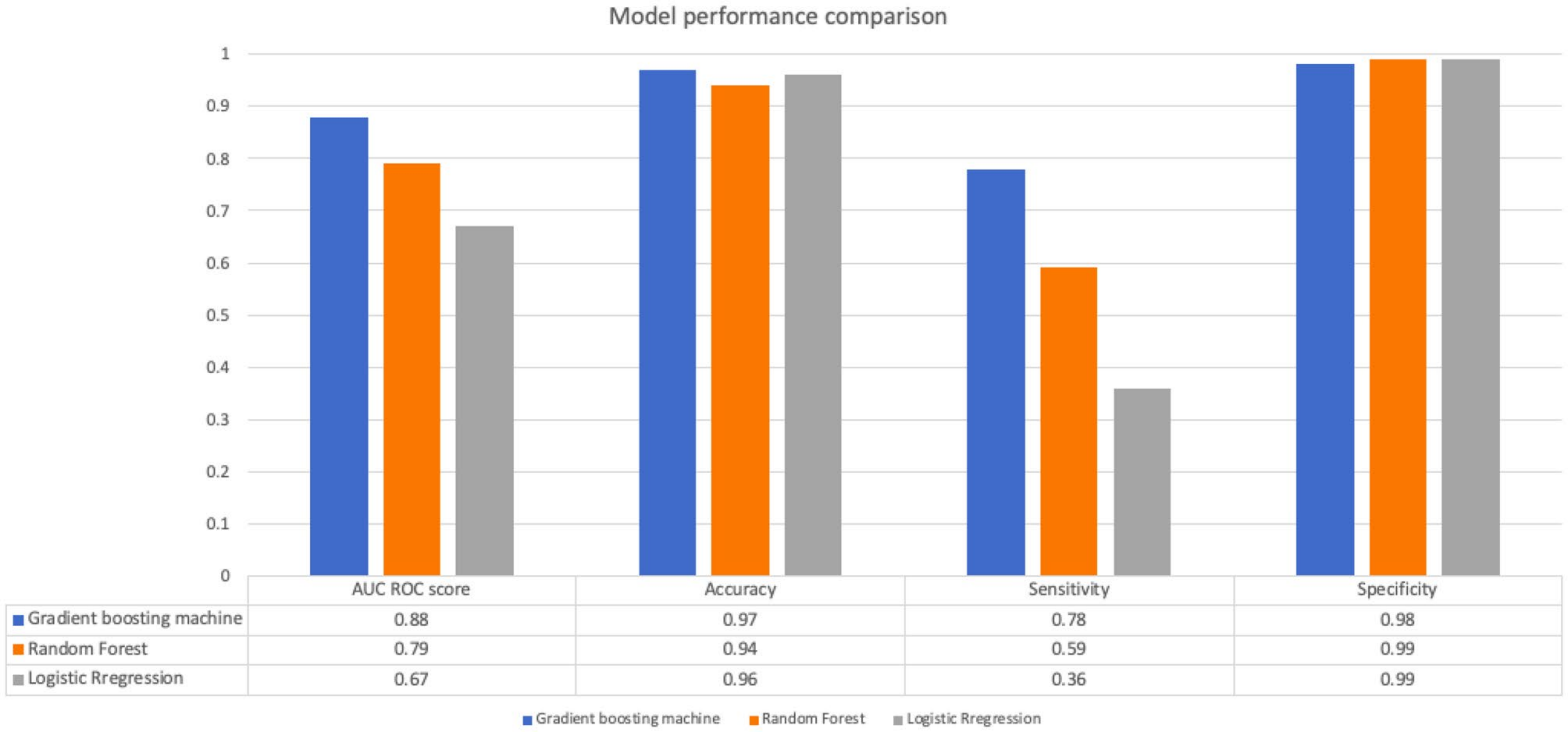
Comparing the performance of eXtreme Gradient Boosting (XGB) Algorithm with Logistic Regression and Random Forest for the development cohort.

When the eXtreme Gradient Boosting (XGB) algorithm is compared with the other models, the comparative results are given in Fig. 4. There is an overall improvement in AUC ROC Scores and Sensitivity in the Gradient Boosting Model compared to the other two models studied.

### Model specification—hazard ratio and survivability plots

We analysed the Hazard Ratios to determine each predictor's effect and contribution in the model and their interplay with other factors. This is done keeping in mind that the odd’s ratios in Table 1 are for single variables where the Hazard Ratios provide the multivariable perspective. The hazard ratios of the 23 variables are provided in Table 2

**Table 2:** Adjusted hazard ratios (multivariate) of the 23 Predictors, calculated based on 28 days Mortality in validation Cohorts.

	Hazard ratio	Confidence interval	z	*p* value
Age > 60 years	2.31	1.52–3.53	3.9	< 0.005
Gender (male)	1.72	1.06–2.85	− 2.2	0.03
Fever	0.28	0.16–0.46	− 4.85	< 0.005
Cough	1.02	0.7–1.47	0.09	0.82
Respiratory distress	1.01	0.62–1.53	0.02	0.84
Weakness	1.69	0.91–2.94	− 1.91	0.05
Diabetes	1.21	0.83–1.77	1	0.14
Hypertension	1.04	0.7–1.55	− 0.16	0.83
Chronic kidney disease	3.04	1.72–5.38	3.88	< 0.005
Chronic liver disease	3.95	1.16–13.42	2.2	0.02
Heart disease	1.56	0.91–2.69	1.61	0.11
ROR	1.54	1.03–2.3	2.03	0.04
Oxygen saturation (SpO2)	2.84	1.87–4.3	2.11	< 0.005
WBC count	1	1	4.9	0.91
Lymphocytes %	1.99	1.23–3.2	− 1.95	< 0.005
INR (prothrombin time)	0.96	0.82–1.13	2.83	0.64
Creatinine	0.82	0.78–0.87	− 0.47	< 0.005
Albumin	1.36	1.03–1.78	− 7.46	0.03
AST (liver enzyme)	1.11	0.89–1.33	1.86	0.01
Lactate dehydrogenase	4.02	2.66–6.07	2.54	< 0.005
Ferritin	2.48	1.32–4.74	6.61	< 0.005
C-reactive protein	1.65	1.11–2.46	− 2.95	0.01
Red cell distribution width	1.03	0.95–1.12	2.45	0.53

**Figure 5:**
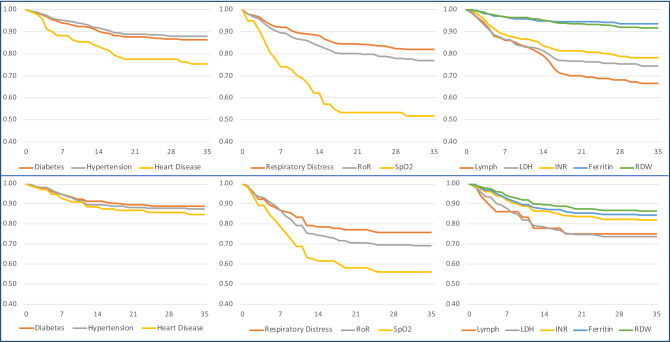
The Graphs compare the KM Plots with the Model (N = 1393) [ABOVE] vs. the Validation (N = 977) [BELOW] Cohorts. The Comorbidities, Respiratory condition at admission, and the significant lab parameters are studied over five weeks for survival from the time of admission. The cut off values for Respiratory Rate—> 24/min; SpO2—< 90%, Lymphocytes—< 10%, LDH > 250 U/L, INR > 1.3, Ferritin > 450 microgm/L and RDW > 14.5% are studied here.

Further to the Hazard Ratios analysis of individual clinical and laboratory predictors, we looked at the predictors' feature in survivability analysis using Kaplan Meier (KM) Plots. KM Plots were prepared for both development and validation cohorts and studied for Comorbidities like Diabetes, Hypertension, and existing Heart Diseases (Coronary Artery Diseases). Heart disease has a seemingly better outcome in the validation cohort, probably due to early intervention and education among patients. In the validation cohort, we can see a considerable change in—Respiratory Distress, Respiratory Rates > 24/min, and Oxygen Saturation below < 90%, which reflects that despite best efforts, patients with significant respiratory failure at admission continue to have a poorer prognosis in 28 days. In Laboratory parameters, LDH, INR, Ferritin, and Red cell Distribution Width show almost similar trends, while low Lymphocyte% contributes higher mortality predominantly in Development Cohort (Fig. 5).

## Discussion

### Interpretation

The AICOVID Risk Prediction Model uses the data from different Indian Hospitals and populations and predicts the outcome (Mortality or Survival) of the patient at the time of admission with 23 Predictors (Clinical and Laboratory) data. This Model, with its existing accuracy, is a comprehensive AI-enabled tool to assist the physician in (a) making triage decision, (b) provide guidance for resource allocation for High and Moderate Risk patients, (c) put them in appropriate Clinical Algorithm (Protocol) Annexure 2 and finally (d) provides a systemic approach of patient and family education related to disease severity during admission. This tool uses basic clinical and laboratory predictors that can be easily accessed in low-cost Indian populations and can be implemented as a secured application programming interface (API).

The model considers Patient’s Age above 60 years as an important predictor and High Risk of Mortality, significantly shown here with higher hazard ratio in the validation cohort (2.31; CI 1.52–3.53)^13^. Interestingly, CDC (Dec 2020) finds similar range of mortality comparison between patients below and above 65 years in US population^14^. Further, male gender has hazard ratios (1.72, 95% CI 1.06–2.85) signifying higher risk of mortality in male population, which is congruent to other international studies^15^ where odds of death has been (1.39; 95% CI 1.31–1.47) comparable. Respiratory Symptoms like distress, higher rate of respiration (> 24/min) or silent hypoxemia detected through lower oxygen level (< 90%) have shown higher risk of mortality (see Table 2) and find their due weightage in the eXtreme Gradient Boosting (XGB) algorithm. The reason to include seemingly similar attributes as they represent different aspects of symptoms and vital physiological parameters at admission. This has also been studied extensively in US patients^16^. The study does not include the High and Moderate Risk patients' subsequent mechanical ventilation and their overall outcome. However, the Kaplan Meir plots (Fig. 5) show outcomes for patients with severe respiratory symptoms at both development and validation cohorts.

The pooled prevalence of Diabetes and Hypertension are 26% and 31.22%, respectively, in the development cohort. The odds of mortality in these comorbid conditions are provided in Table 1, comparable with the meta-analysis conducted by Kumar et al.^17^. Hazard ratios associated with Chronic Kidney Disease (3.04, CI 1.72–5.38) (Prevalence 6.2%) shows the higher associated risk with comparable results from ERA-EDTA Council and the ERACODA Working Group^18^. The prevalence of Chronic Liver Disease (3.95, CI 1.16–13.42) was comparatively lower than other comorbidities, and hence hazard ratio is higher than other studies^19^. Hazard associated Pre-existing Coronary Artery Disease (1.56, CI 0.91–2.69) (Prevalence 7.3%) showed a lesser hazard ratio probably due to timely intervention and management, compared to studies by Cheng Y et al. and China CDC^20,21^.

In lab features conducted during admission, lymphopenia (Lymphocyte < 12%) had a hazard ratio (1.99, CI 1.23–3.2), which is consistent with the systemic review and meta-analysis^22^. Other significant factors included pro-inflammatory markers like LDH (cut off—> 250 U/L), Ferritin (cut off—> 450 µg/L), and CRP (cut off—> 48 mg/L) (See Hazard Ratio—Table 2). This is consistent with various international studies with slightly modified cut-off values^23–25^. Though the study did not attribute different features like Neutrophil—Lymphocyte Ratio or CRP / Albumin Ratio, it did look into the Hazard Ratios separately^25^.

**Figure 6:**
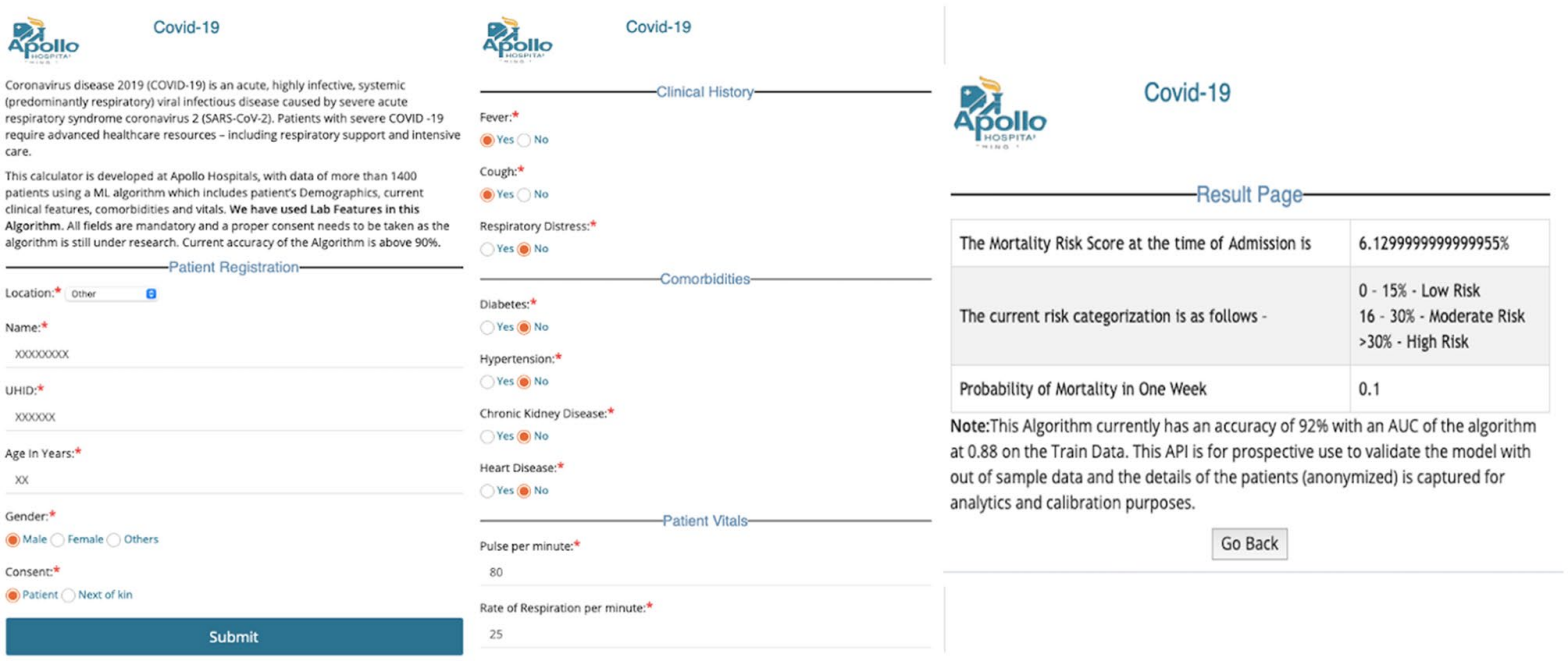
The API Generated from the Algorithm which provides the Risk Score for dummy data on their Clinical and Lab Parameters at admission (done for quality assurance purpose).

As discussed above, the model takes all the above features, reasonably congruent with the published international studies, and provides the prediction using an eXtreme Gradient Boosting (XGB) method. The eXtreme Gradient Boosting (XGB) method is a popular technique that recursively fits multiple predictors and a weak learning system with the residual to increase the model's accuracy using various iterations^26^. The model inherently identifies the complex data structure—including their interaction and nonlinearity in the context of multiple predictors. For further use, the model would be calibrated with the data provided in the API in Fig. 6 (seen here with dummy data for quality assurance purpose) and continuously improve over a period of time. The Risk Model is available for clinicians at http://20.44.39.47/covid19v2/page1.php.

### Implication

All measures have been taken to reduce bias, including socio-economic aspects, as Apollo Hospitals admitted patients from all society sections with moderate to severe diseases. Potential clinical use of the Clinical API developed from the AICOVID Algorithm is currently being used in Apollo Hospitals. It can be used in the Indian Subcontinent hospitals with the current accuracy and precision. In the prospective validation study, patient written consents were obtained through an appropriately designed consent form (Annexure 4), paving the way for further research of using written consent in Clinical AI-enabled tools and their appropriate patient information.

The developed model—AICOVID and its corresponding Application—is being utilized for triaging, allocating resources, putting patients into an appropriate clinical protocol, and patient and family education at admission. This has reduced time to appropriate bed allocation and Isolation ER holding times. Further, it has helped in reducing cost in ordering necessary tests at admission, thereby using patient’s and hospitals’ resources at optimum.

Similar models have been created at MIT Sloan School^10^ and Tencent AI Lab^27^ using 7 and 10 clinical features and similar accuracy and precision. However, these tools are more specific and accurate to the population whose data is being used to build the models. The steps of the research is conducted in accordance with TRIPOD Checklist^28^.

## Limitation

The model is prepared with Clinical and Laboratory features that are available at the time of admission in Emergency Room or other clinical settings. One of the study's limitations is that it does not include imaging tests done at the time of admission, as many of the critical patients couldn’t undergo imaging tests within first 6 h of admission. Due to logistical issues, certain clinical parameters like Body Mass Index (BMI) and follow-up information on patient’s ventilation details were not obtained. Furthermore, due to the unavailability of adequate data—certain laboratory markers like D-dimer and Interleukins were excluded. The research team is currently undertaking the analysis of the follow-up care of these patients (survivors).

Geographical and Ethnic acceptance—The Apollo Hospitals included in the study are from Bangalore Chennai, Delhi, Hyderabad, Kolkata, and Navi Mumbai. This provides comprehensive coverage of the Indian population, looking at possible zones. However, further studies are required for validation and calibration purposes in Western and Eastern (including North Eastern) zones. Beyond India, further research is needed to calibrate the model when used in other population like the US, Europe, Middle East, and South East Asia.

## Conclusion

This study and model on mortality risk prediction provides insight into the COVID Clinical and Laboratory Parameters at admission with an accuracy of 97% at Development and 93% at Validation Cohort respectively. The model is built to ensure that it can be used in low cost settings to improve triage and resource allocation. This is one of the initial studies reflecting on ‘time to event’ at admission, accurately predicting patient outcomes, done on the Indian population. Future studies should include a global approach with inclusion of Imaging parameters.

## Supplementary information

Supplementary Informations.

## Data Availability

Data cannot be shared with any 3rd party.
