# Supplementary material for: Multivariable mortality risk prediction using machine learning for COVID-19 patients at admission (AICOVID)
Source: Sci Rep. 2021 Jun 17;11:12801. doi: 10.1038/s41598-021-92146-7 (PMC8211710; doi:10.1038/s41598-021-92146-7)
Supplement: Supplementary file 1 — Supplementary Informations. [file 41598_2021_92146_MOESM1_ESM.docx]

Title: Multivariable Mortality Risk Prediction using Machine Learning for COVID-19 patients at admission (AICOVID)

Kar S, Chawla R, Haranath SP, Ramasubban S, Ramakrishnan N, Vaishya R, Sibal A, Reddy S

Annexure 1 – Patient Clinical Data Collection Format

| **Sr. No.** | **Case number** |  |
| --- | --- | --- |
|  | **UHID Number** |  |
| 1 | Date of Admission |  |
| 2 | Was it a direct admission – Yes/ No |  |
| 3 | Was the patient admitted in any other hospital - Yes / No if yes, please mention the details |  |
| 4 | Date of Discharge |  |
| 5 | Age |  |
| 6 | Sex |  |
| 7 | History and Chief complaints |  |
| 8 | History of present illness at the time of reporting to the hospital |  |
| 9 | Co-morbidities if any (Diabetes mellitus, hypertension, chronic kidney/liver/lung/heart disease, immune suppression/ PLHIV) |  |
| 10 | Vitals (on admission) | Pulse (Heart rate)- Blood Pressure- Temperature Respiratory rate |
| 11 | SpO2 levels (background FIO2) on admission |  |
| 12 | Date of sample sent for COVID test |  |
| 13 | Date of patient declared positive for COVID-19 |  |
| 14 | Onset of Respiratory Failure (Non-Life Threatening) |  |
| 15 | Onset of Respiratory Failure (Life Threatening) |  |
| 16 | Risk of Exposure to Non-Covd Designated Staff? |  |
| 17 | Category (Please tick the category) - refer to "Investigation and treatment protocol for COVID-19" of Apollo |  |
| 18 | Category A- Mild |  |
| 19 | Category B-1 |  |
| 20 | Category B-2 - Moderate |  |
| 21 | Category C- Severe |  |
| 22 | Whether admitted to ward or ICU |  |
| 23 | Tests carried out with results |  |
| 24 | 1 FERRITIN- 396.3 |  |
| 25 | 2 INTERLEUKIN 6 -12.0 pg/ml |  |
| 26 | 3BUN -10 mg/dL |  |
| 27 | 4 C-REACTIVE PROTEIN (CRP) 15.2 mg/L |  |
| 28 | 5 D-DIMER 0.35 µg/ml |  |
| 29 | Whether CT chest (non contrast) done or not (if yes then please mention findings) |  |
| 30 | Treatment given (mention name of medication with doses) |  |
| 31 | Was an ECG done daily or not |  |
| 32 | Any experimental Therapy tried or not. If no Experimental therapy was given, kindly share the reasons |  |
| 33 | Whether Plasma therapy was done or not |  |
| 34 | Was the patient treated as per Apollo Porotcol? Yes or No |  |
| 35 | Deviation of treatment from Apollo protocol? Yes or No |  |
| 36 | Use of NIV |  |
| 37 | Date of start of ventilation |  |
| 38 | Threshold of putting on invasive ventilation |  |
| 39 | Whether any delay in diagnosis (Yes/ No) |  |
| 40 | Whether any delay in treatment (Yes/ No) |  |
| 41 | Speciality of the Primary Consultant |  |
| 42 | Whether the patient was intubated or not |  |
| 43 | Mention if any other specialist was involved in treatment |  |
| 44 | Need of additional life supportive means |  |
| 45 | Any other relevant information |  |
| 46 | Please mention the total number of discharges till date for which information has been sent |  |
| 47 | **Instructions** |  |
| 48 | A. Please share the details of each recovered discharge cases in the above format B. Please mention the case number in the serial order and UHID number  C. Kindly do not write IP number or name of patient.  D. In case you are sending the scanned copy the discharge summay then kindly hide the name and IP number black out with a black marker E. Kinldy report all discharged cases during the period Saturday to Friday of each week by Saturday |  |

Annexure 2


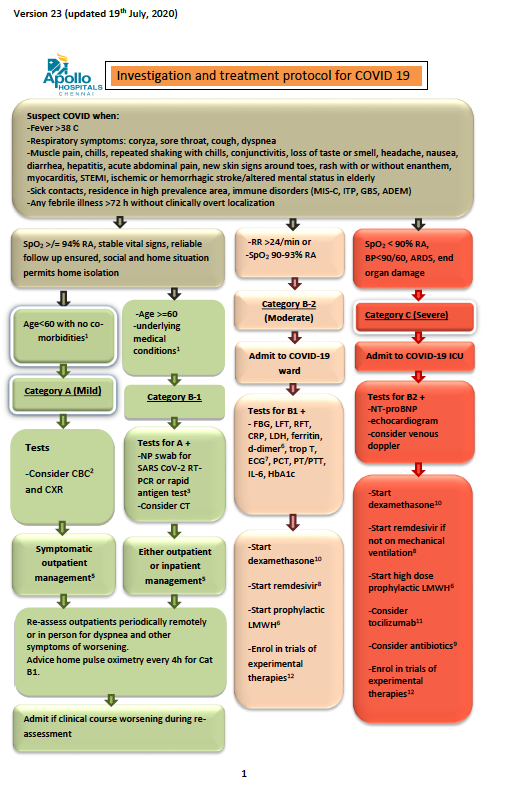


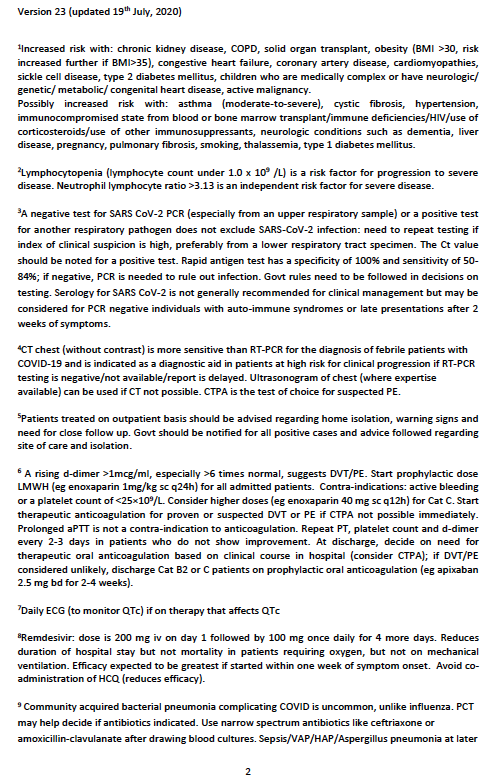


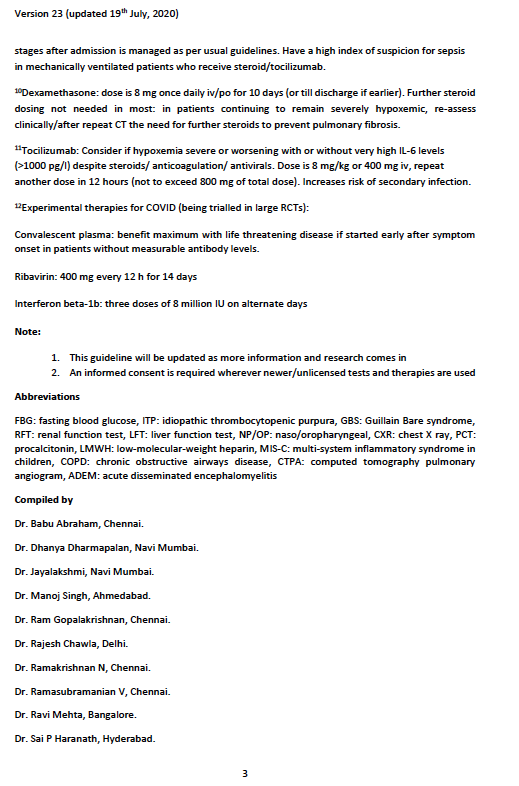


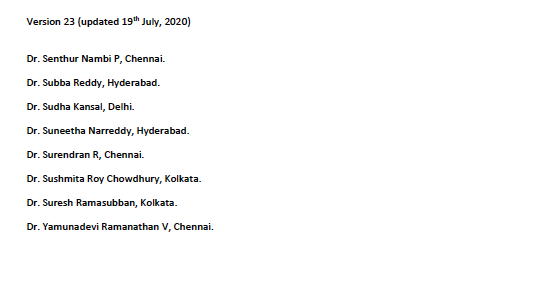


Annexure 3

We considered the XGB model, as the function of this model is an approximation of the data distribution considering the errors:

$$y_{i}=F_{1}\left( X_{i} \right)+\mathrm{error}_{1i}$$

Where $y_{i}$ is the predicted value and $X_{i}$ are the input values. $F_{1}(x_{i})$ is a function, and the relationship between $X$ and $y$ is not fully described.

1. We initialize the model by solving the following equation for the 23 input parameters :

$F_{0}(x)=\arg\min\sum_{i=1}^{n} L(y_{i},\gamma$); then we get

$$F_{0}\left( x \right)=\frac{\sum_{i=1}^{n} y_{i}}{n}$$

Where $n$ is the total number of observation, i.e., 1393. $F_{1}(x_{i})$ is function a weak learner, and the relationship between X and y is not fully described

1. For no of iterations - m = 1 to M

Gradient with respect to predicted value,

$$r_{\mathrm{im}}= -\left\{ \frac{\partial L\left[ y_{i},F_{m-1}\left( x_{i} \right) \right]}{\partial F_{m-1}\left( x_{i} \right)} \right\},$$

Where $i$ is the index for observations $m$ represents the number of iterations with m ∈ [1, M].

1. Fit the weak learner $h_{m}(x)$ to the residuals by:

Computing the $\gamma_{m}$ to solve the optimization problem:

$\gamma_{m}=\arg\min\sum_{i=1}^{n} L[y_{i},F_{m-1}\left( x_{i} \right)+.F_{m}(x_{i})]$

By solving this equation we can get:

$$\gamma_{m}= \frac{\sum_{i=1}^{n} h_{m}(x_{i})\cdot[y_{i}-F_{m-1}\left( x_{i} \right)]}{{\sum_{i=1}^{n} h_{m}(x_{i})}^{2}}$$

1. Update the $F_{m}\left( x \right)= F_{m-1}\left( x \right)+ \gamma_{m}\cdot h_{m}(x)$

squared error is used as the loss function, and the gradient of the loss function can be calculated as follows:

$$r_{\mathrm{im}}= -\left\{ \frac{\partial L\left[ y_{i},F_{m-1}\left( x_{i} \right) \right]}{\partial F_{m-1}\left( x_{i} \right)} \right\},$$

$$= - \frac{\partial\{\frac{1}{2}\times[F_{m-1}\left( x_{i} \right)-y_{i}]^{2}\}}{\partial F_{m-1}(x_{i)}}$$

$$=-\frac{\partial\{\frac{1}{2}\times\left[ F_{m-1}\left( x_{i} \right)^{2}+y_{i}^{2}-2\times F_{m-1}\left( x_{i} \right)\cdot y_{i} \right]\}}{\partial F_{M-1}(x_{i})}$$

=$y_{i}-F_{m-1}\left( x_{i} \right) for i=1,2,\ldots n.$

Annexure 4 –

INFORMED CONSENT DOCUMENT

Patient Information sheet and Informed Consent Form

PART A: STUDY PARTICIPANT INFORMATION SHEET

STUDY TITLE: Development and Validation of a multivariable prediction model using Machine Learning to predict the outcome of admitted COVID patients at admission

INSTRUCTIONS:

Please read and understand the information given below. While reading the document and during the course of the study, you are free to ask any study related question. Your question will be answered and required study related clarification will also be provided. LAR (Legally Acceptable Representative. A signed copy of this document will be provided to you.

1. Introduction: You are being invited to take part in the research study which is done as a part of identifying risk factors for Coronavirus Disease in Indian Population. The details of the study are described below for your understanding. If you have any queries/clarifications feel free to ask me.
2. Background:

Overview: Coronavirus disease 2019 (COVID-19) is an acute, highly infective, systemic (predominantly respiratory) viral infectious disease caused by severe acute respiratory syndrome coronavirus 2 (SARS-CoV-2). Identified in December 2019 in Wuhan, China, and has led to an ongoing pandemic and as of 28 July 2020, more than 16.4 million cases have been reported across 188 countries and territories, resulting in more than 654,000 deaths. In India, as of July 28, 2020, there are over 1.48 Million cases, with nearly 1 Million recovered and over 33,500 people dead. There is a need to conduct a study project on patient data to determine, & interpret risk factors associated with Coronavirus Disease in Indian Population.

Goal: The goal of the study is to determine relevant Risk Factors associated with Coronavirus Disease, develop and validate COVID -19 risk score prediction model and integrate this in Healthcare Organizations’ Electronic Medical Record

Methodology: The retrospective data analysis arm of the study included 1435 participants aged between 0 to 80 between year from March 1 to July 20, 2020 from 8 centres of Apollo Hospitals. A multi-step risk factors selection process was used to build the prediction model. The model was built with clinical and lab parameters. The machine learning techniques used were Binary Fit Logistic Regression, Classification and Regression Tree and combination of Cox Proportional Hazard Model with Gradient Boosting System.

Inferences: Study is consistent with International Studies on higher significance for risk factors like higher age group (> 50 years) diabetes, hypertension, respiratory distress, vitals like rate of respiration, oxygen saturation, critical care admission, blood parameters like low lymphocytes and eosinophils, and increase in pro – inflammatory markers.

Conclusion: This is the first Electronic Medical Record Integrated COVID Risk Score related project and risk model on Indian population using retrospective & prospective data which can be helpful in better prediction and management of Coronavirus disease. The tool also integrates a Clinical Protocol designed and devised by a panel of Apollo Hospitals Consultants following the Risk Score generation which would provide a proper approach for further management.

1. The Purpose of the study

To determine a Standardized Risk Score for Coronavirus Disease

2. Who can take part in the study?

Any individual

- Aged between 18-79 years
- Willing to provide informed consent and comply with study procedure
- Presenting with signs and symptoms of Coronavirus Disease like Fever, Cough, Difficulty Breathing (Respiratory Difficulty), Headache, Body ache, Weakness or other symptoms
- Asymptomatic patients with Comorbidities like higher age group, Diabetes, Hypertension, Chronic Kidney Disease, Cancers, Coronary Artery Disease etc.

3. Duration of Your participation

You will be followed up for a period of 1 year.

4. Total No. of study participants:

The total number of study participants are 2400

5. Possible benefits of taking part in this study:

1. Understand the different Coronavirus Risk Factors
2. Determine your current and prospective scores (during the stay at the hospital)
3. Determine your risk factors and how this can be addressed through appropriate therapeutic intervention and management

6. Possible disadvantage or risks of taking part in this study:

1. 1 year periodic follow up for discharged patients

7. Study procedure:

During the screening and assessment at admission, your Investigator (doctor) shall review the following information as part of the routine medical/clinical care –

1. Age: Your Age in years.
2. Gender: Your Gender - Male/Female.
3. Symptoms :
   1. Travel and Contact History
   2. Fever, Cough, Difficulty Breathing (Respiratory Difficulty), Headache, Body ache, Weakness or other symptoms
   3. Duration of illness
4. Comorbidities like Diabetes, Hypertension, Chronic Kidney Disease, Cancers, Coronary Artery Disease etc.
5. Vitals
   1. Temperature : In degree Fahrenheit
   2. Pulse Rate : Beats per minute
   3. Systolic Blood Pressure: The blood pressure when the [heart](https://www.medicinenet.com/script/main/art.asp?articlekey=3668) is contracting.
   4. Diastolic Blood Pressure: The diastolic pressure is specifically the minimum arterial pressure during relaxation and dilatation of the [ventricles](https://www.medicinenet.com/script/main/art.asp?articlekey=5984) of the heart when the ventricles fill with blood.
   5. Rate of Respiration: Number of Breaths per Minute.
   6. Oxygen Saturation or SpO2 (as commonly known) : It is a measure of the amount of oxygen-carrying hemoglobin in the blood relative to the amount of hemoglobin not carrying oxygen.
6. Admission to ICU (Critical Care) : Depending on your condition and the process of triage, the Principal Investigator or treating team may choose to admit you in the ICU (Critical Care)
7. Laboratory Parameters : To determine your current condition, the Principal Investigator or treating team shall order few common and advanced tests. Some of them are enumerated below –
   1. Complete Blood Count : Haemoglobin, Total White Blood Cell Count (WBC), Differential Leucocyte Count (DLC), Platelet Count
   2. Biochemical Tests : Liver Function Tests including Albumin, Transaminases (AST or ALT), Creatinine and Glycosylated Haemoglobin (HbA1c)
   3. Coagulation Tests : Prothrombin Time, Activated Partial Thromboplastin Time, International Normalized Ratio (INR) etc.
   4. Proinflammatory Markers : C – reactive Protein, Creatine Kinase, D-dimer, Ferritin, Interleukin – 6, Lactate dehydrogenase, and Procalcitonin.
   5. Electrolytes : Sodium, Potassium, Magnesium, Bicarbonates and Chloride.

8. Collection of data:

Your data shall be collected in a standardized electronic format

9. Confidentiality of data :

Confidentiality

The organization and the Investigator shall maintain the confidentiality of data, especially with respect to the information about the patient or any other individual sharing their health information, which would otherwise be known only to the physician or care givers. The data shall be secured & stored in the Electronic and Manual Medical Records.

Data Content

Your data shall include the demographic & registration details, clinical data – personal & family history, assessment details, treatment and medication details, investigations like lab diagnosis, diagnostic imaging and other invasive procedures.

Source of Data

The source of your data shall include the current records, results of the assessment and diagnostic & treatment procedures. The data are generated and shared with the organization during the processes of registration, consultation and ongoing treatment at the different locations in the organization.

Data sharing for research purposes

The organization and the Investigator shall use your data, following anonymization and de – identification of the individual for retrospective and prospective study purposes.

Conformation to Laws & Regulation

The organization and the Investigator shall conform to the national laws and internal guidelines on collection, security & storage and sharing of the data – Indian Council of Medical Research, NDCT clinical trials rule 2019, & Drug Controller General of India guidelines, General Data Protection Regulation 2016 (European Union), World Health Organization and Food and Drug Administration (United States). The Institutional Ethics Committee of respective research sites, where data are used and analyzed for research purpose, are accredited by National Accreditation Board for Hospitals and Healthcare Providers (India).

Data Sharing with other Organizations

Data Sharing with other Organizations - The organization and the Investigator shall not share data with other organizations unless required under law and following appropriate contract to institutions, where the data isn’t stored in facilities or servers outside the country abiding by the national laws. All data are appropriately encrypted meeting necessary specification and shared with anonymity

Withdrawal of Consent for Sharing Data

You may choose to withdraw your consent to share your data for research purposes which would be respected by the Organization and the Investigator

10. Signing of the consent form and keeping a copy of the Patient Information Sheet:

On agreeing to participate in the study, you may sign the consent form with your legally accepted representative. The investigators shall provide a copy of the signed Patient Information Sheet and Informed Consent Form to Study Subjects

11. What will happen to the results of the research/trial study?

The results of the study shall be published and shall be available for public use as Standardized Coronavirus Risk Score for Indian Population. You will not be identified in any report/publication.

12. Who is organizing the study? Are there any compensation for participation?

The observational study is organized Apollo Hospitals. There are no financial compensation for participation, travel, laboratory, diagnostic tests or disease management

As this study is an observational, prospective data collection study, there are no allocated compensation. You would have to pay for the visit to the investigator, including your tests and investigations, travel to the hospital or clinic, any further medication or intervention required for your treatment.

24. Who has reviewed the study?

The Institutional Ethics Committee – Bio Medical Research [IEC-BMR] Apollo Hospitals ----------- has reviewed and approved the study

25. Contact for further information.

For any further information / questions, you and/or your LAR can contact any of the following for the purposes mentioned below:

| 1. For Queries Related to the Study | | |
| --- | --- | --- |
| Role in the Study | Name | Contact Details |
| Study Investigator (s) |  |  |
| 1. For Queries Related to Your Rights as a Participant in a Clinical Study | | |
| Chairperson, |  |  |
| Member Secretary, Ethics Committee |  |  |
| 1. For Making a Claim for Financial Compensation in case of Study Related Injury or Death – NOT APPLICABLE | | |
| Sponsor |  |  |

| Title of the Study |  | | | | | | | |
| --- | --- | --- | --- | --- | --- | --- | --- | --- |
| Study Number |  | | | | | | | |
| Subject’s complete Name |  |  | |  | | |  | |
|  | Title | First Name | | Middle Name | | | Surname | |
| Subject’s Initial |  | | | | | | | |
| Date of Birth (dd/mmm/yyyy) |  | | | Age (in years) | |  | | |
| Subject’s Address (complete with city and zip code) | Address: | | | | | | | |
|  | Phone No. (with STD Code):  Mobile No.: | | | | | | | |
| Subject’s Qualification (please mention complete degree) |  | | | | | | | |
| Subject’s Occupation  (please tick box as appropriate) | Student Self-employed  Service Housewife  Others: ……………………………… | | | | | | | |
| Subject’s Annual Income (in INR) |  | | | | | | | |
| Nominee’s Complete Name  (an individual or entity who is nominated by the subject for the purpose of receiving compensation in case of study related death) |  | |  | |  | | |  |
|  | Title | | First Name | | Middle Name | | | Surname |
| Nominee’s relationship to the Subject |  | | | | | | | |
| Nominee’s Address and Contact Details  (complete with city and zip code) | Address: | | | | | | | |
|  | Phone No. (with STD Code):  Mobile No.: | | | | | | | |

11. Questions:

Part B: CONSENT TO ACT AS A PARTICIPANT IN THE STUDY

Study Title:

| Name of Patient |  | Patient Initials |  |  |  |
| --- | --- | --- | --- | --- | --- |
| Age (years) |  | Screening No. |  |  |  |

(Participant initials / signature / left thumb impression)

1. I confirm that I have read and understood the information sheet for the above observation study and have had the opportunity to ask questions
2. I understand that my participation in this study is voluntary and that I am free to withdraw at any time without my medical care or legal rights being affected and without giving any reason
3. I understand that the ethics committee and the regulatory authorities will not need my permission to look at my health records both in respect of the current study and any further research that may be conducted in relation to it, even if I withdraw from the study. I agree to this access. However, I understand that my identity will not be revealed in any information released to third parties or Published.
4. I understand that my identity will not be disclosed in any information released to any third parties or published.
5. I agree not to restrict the use of any data or results resulting from this research study provided such a use is only for scientific purpose(s)
6. I agree to take part in the above data study.

Signature of Participant Date

(If the participant is illiterate then left thumb impression)

Signature of Legally Accepted Representative Relation to participant

(LAR)

Signature of Witness Date

(If LAR is illiterate please provide witness signature)

Name of Investigator / Designee Signature Date
